# Increase in Ethanol Yield via Elimination of Lactate Production in an Ethanol-Tolerant Mutant of *Clostridium thermocellum*

**DOI:** 10.1371/journal.pone.0086389

**Published:** 2014-02-07

**Authors:** Ranjita Biswas, Sandeep Prabhu, Lee R. Lynd, Adam M. Guss

**Affiliations:** 1 Biosciences Division, Oak Ridge National Laboratory, Oak Ridge, Tennessee, United States of America; 2 BioEnergy Science Center, Oak Ridge National Laboratory, Oak Ridge, Tennessee, United States of America; 3 Department of Bioengineering, University of California Berkeley, Berkeley, California, United States of America; 4 Thayer School of Engineering at Dartmouth College, Hanover, New Hamphire, United States of America; Virginia Commonwealth University, United States of America

## Abstract

Large-scale production of lignocellulosic biofuel is a potential solution to sustainably meet global energy needs. One-step consolidated bioprocessing (CBP) is a potentially advantageous approach for the production of biofuels, but requires an organism capable of hydrolyzing biomass to sugars and fermenting the sugars to ethanol at commercially viable titers and yields. *Clostridium thermocellum*, a thermophilic anaerobe, can ferment cellulosic biomass to ethanol and organic acids, but low yield, low titer, and ethanol sensitivity remain barriers to industrial production. Here, we deleted the hypoxanthine phosphoribosyltransferase gene in ethanol tolerant strain of C. thermocellum adhE*(EA) in order to allow use of previously developed gene deletion tools, then deleted lactate dehydrogenase (*ldh*) to redirect carbon flux towards ethanol. Upon deletion of *ldh*, the adhE*(EA) Δ*ldh* strain produced 30% more ethanol than wild type on minimal medium. The adhE*(EA) Δ*ldh* strain retained tolerance to 5% v/v ethanol, resulting in an ethanol tolerant platform strain of *C. thermocellum* for future metabolic engineering efforts.

## Introduction

A major challenge of this century is to develop sustainable technology for production of fuels and chemicals independent of fossil fuels. Lignocellulosic biomass is an abundant resource [1] that has potential to be used as a feedstock for the production of fuels and chemicals. Consolidated Bioprocessing (CBP) [2], [3] is a promising approach that could help make cellulosic fuel production economical; however, no natural organisms are known that can both hydrolyze cellulosic biomass and produce a liquid fuel at high yield and titer under industrially relevant conditions.

*Clostridium thermocellum* is a cellulolytic bacterium that has potential to be engineered for CBP. It is known to rapidly ferment cellulosic biomass to ethanol, acetate, formate and lactate. But unlike highly ethanologenic microbes such as *Saccharomyces cerevisiae* and *Zymomonas mobilis*, wild type strains are only able to tolerate low levels of ethanol (ca. 20 g/L) [4]. Increased tolerance would allow higher titer and more economic product recovery. Recent studies have aimed to understand the mechanisms by which *C. thermocellum* is able to evolve tolerance to higher levels of ethanol [5], [6]. An ethanol tolerant strain of *C. thermocellum* ATCC 27405 was developed to tolerate ethanol concentration up to 5% v/v [6]. Genome sequencing of this ethanol tolerant strain revealed approximately 400 mutations. Amongst these mutations, two single nucleotide polymorphisms were found in the alcohol dehydrogenase (*adhE*) gene [7]. Transfer of this mutated gene to *C. thermocellum* DSM 1313 conferred ethanol tolerance, resulting in strain C. thermocellum adhE*(EA). Cell extracts from both the original ethanol tolerant mutant and adhE*(EA) contained no detectable NADH-dependent alcohol dehydrogenase (ADH) activity and an increase in the NADPH-dependent ADH activity, suggesting a link between ethanol sensitivity, central metabolism and redox cofactor balancing [7].

The C. thermocellum adhE*(EA) strain decreased ethanol and increased lactate synthesis relative to wild type *C. thermocellum* DSM 1313 [7]. To begin to understand the metabolic effects of altered ADH cofactor specificity and to build a platform strain for future metabolic engineering, we deleted the hypoxanthine phosphoribosyltransferase gene (*hpt*) to create a genetic background for making unmarked gene deletions [8]. The redirection of carbons and electrons as lactate in ethanol tolerant C. thermocellum adhE*(EA) strain offered a metabolic challenge to understand ethanol tolerance and improve ethanol production. In this study, lactate dehydrogenese (*ldh*) was deleted in an attempt to redirect carbon and electrons towards the ethanol production pathway.

## Materials and Methods

### Strains and Culture Conditions

*Escherichia coli* TOP10 and BL21 were grown in LB medium supplemented with 12 µg ml^−1^ chloramphenicol when appropriate. *Clostridium thermocellum* DSM1313 and mutant strains were grown in the rich medium described by Tripathi [9], which is based on DSM122 medium, supplemented with 5 µg ml^−1^ thiamphenicol, 50 µg ml^−1^ 5-fluoro-2′-deoxyuridine and 500 µg ml^−1^ 8-azahypoxanthine as needed. Final medium composition was (L^−1^): 3 g sodium citrate tribasic dehydrate, 1.3 g ammonium sulfate, 1.43 g potassium phosphate monobasic, 1.8 g potassium phosphate dibasic trihydrate, 0.5 g cysteine-HCl, 10.5 g 3-morpholino-propane-1-sulfonic acid (MOPS), 6 g glycerol-2-phosphate disodium, 5 g cellobiose, 4.5 g yeast extract, 0.13 g calcium chloride dehydrate, 2.6 g magnesium chloride hexahydrate, 0.0011 g ferrous sulfate heptahydrate and 0.0001 g resazurin, adjusted to pH 7.0. The minimal medium used was MTC [10] prepared as described in [5], consisting of (L^−1^): 2 g sodium citrate dehydrate, 1.25 g citric acid monohydrate, 1 g sodium sulfate, 1 g potassium phosphate dibasic trihydrate, 2.5 g sodium bicarbonate, 1.5 g ammonium chloride, 2 g urea, 1 g magnesium chloride hexahydrate, 0.2 g calcium chloride dehydrate, 0.1 g ferrous chloride tetrahydrate, 1 g L-cysteine hydrochloride monohydrate, 5 g cellobiose, 0.001 g resazurin, 5 g 3-morpholino-propane-1-sulfonic acid (MOPS), 20 mg pyridoxamine dihydrochloride, 1 mg riboflavin, 1 mg nicotinamide, 0.5 mg DL-thioctic acid, 4 mg 4-amino benzoic acid, 4 mg D-biotin, 0.025 mg folic acid, 2 mg cyanocobalamin, 4 mg thiamine hydrochloride, 0.5 mg MnCl_2_.4 H_2_O, 0.5 mg CoCl_2_.6 H_2_O, 0.2 mg ZnSO_4_.7 H_2_O, 0.05 mg CuSO_4_.5 H_2_O, 0.05 mg HBO_3_, 0.05 mg Na_2_MoO_4_.2 H_2_O, 0.05 mg NiCl_2_.6 H_2_O.

### Plasmid and Strain Constructions

To delete *hpt* for use as a negative selectable marker as in [8], plasmid pAMG231 (sequence in File S1) was transformed in C. thermocellum adhE*(EA) via electroporation as previously described [11]. Deletion of the chromosomal copy of *hpt* (Clo1313_2927) and simultaneous plasmid loss was selected using 500 µg ml^−1^ 8-azahypoxanthine (Acros Organics, Pittsburgh, PA), as previously described [8], generating strain C. thermocellum adhE*(EA) Δ*hpt*. Plasmid pMU1777 was used as previously described [8] to delete *ldh* (Clo1313_1160), resulting in strain C. thermocellum adhE*(EA) Δ*hpt* Δ*ldh*, which is hereafter referred to as C. thermocellum adhE*(EA) Δ*ldh*. Deletion was confirmed by PCR as described in the text, using primers P1- forward TATTATTTCTTTTAGAGTGTTTCCGG, P1/P2- Reverse CATTGATGCTCAGCGGACTT, P2-forward- CGTACTGTCCTTCCAAAAGGC, P3-forward- CTTGGCTTCATTGCTGTAAGATAC, and P3- reverse- ACCGCTGGAACATTAACAGATT. The strain was further confirmed by 16S rRNA gene sequencing and sequencing of the relevant chromosomal mutations in adhE*(EA).

### Fermentation Conditions

The inoculum for batch fermentation was prepared by growing the mutants in rich medium overnight at 55°C in an anaerobic chamber (COY laboratory products, Inc.). The fermentation was carbon limited and carried out in 25 ml Balch tubes with 10 ml of either rich or minimal media containing 5 g L^−1^ of cellobiose under a headspace of 20∶80% v/v CO_2_:N_2_ mixture sealed with butyl rubber stoppers. The tubes were inoculated with 0.5% v/v culture and incubated at 55°C. The samples from the tubes were collected at regular intervals using 1 ml syringe to determine the fermentation product concentrations. The fermentation products were determined after 48 h of growth.

### Ethanol Tolerance

Ethanol tolerance was tested in Balch tubes containing rich medium with 0, 1, 2, 3, 4 and 5% v/v added ethanol, inoculated with 0.5% of overnight grown culture and incubated at 55°C. Growth was monitored by measuring the optical density at 600 nm on a Unico 1200 spectrophotometer.

### Analytical Methods

Fermentation products including ethanol, acetate, lactate and formate were analyzed on Breeze 2 HPLC (High Performance Liquid Chromatograph) system using an Aminex-HPX-87H column with a 5 mM sulfuric acid mobile phase.

## Results

### Deletion of Lactate Dehydrogenase in C. thermocellum adhE*(EA) Increases Ethanol Production

**Figure 1:**
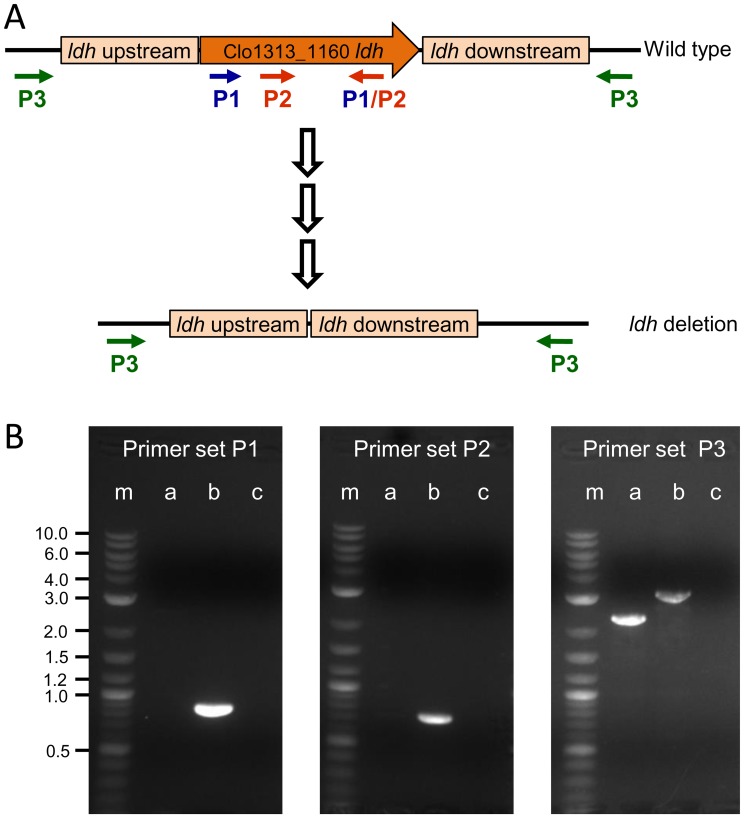
Deletion of *ldh* – overview and confirmation. A) The *ldh* gene was deleted using the same methodology as [8]. Primer binding sites for PCR detection of *ldh* are indicated with arrows. P1, binding sites for forward and reverse primer set 1, P2, binding sites for forward and reverse primer set 2 and P3, binding sites for forward and reverse primer set 3. B) PCR confirmation of deletion of lactate dehydrogenase (*ldh*). Lane m, DNA ladder with molecular weights noted (in kilobases); Lane a, adhE*(EA) Δ*ldh* template; Lane b, *C. thermocellum* wild type template; Lane c, No template PCR control.

To better understand metabolic flux and to build a platform strain for further metabolic engineering, hypoxanthine phosphoribosyltransferase (*hpt*) was deleted to provide a counter-selectable marker for further genetic manipulations, followed by deletion of the lactate dehydrogenase gene (*ldh*) from the chromosome of C. thermocellum adhE*(EA). Three primer sets were used to confirm the *ldh* gene deletion in the wild type locus (Fig. 1A and B). Primer set (P1) and (P2) amplified 863 bp and 729 bp fragments of the *ldh* gene, respectively, which is present in the wild type but absent in adhE*(EA) Δ*ldh*. Primer set (P3) amplified the 3200 bp region of the wild type locus, while amplification from adhE*(EA) Δ*ldh* resulted in a 2200 bp fragment, confirming gene deletion. The *adhE* gene was further sequenced to verify that the strain maintained the adhE*(EA) mutations.

**Figure 2:**
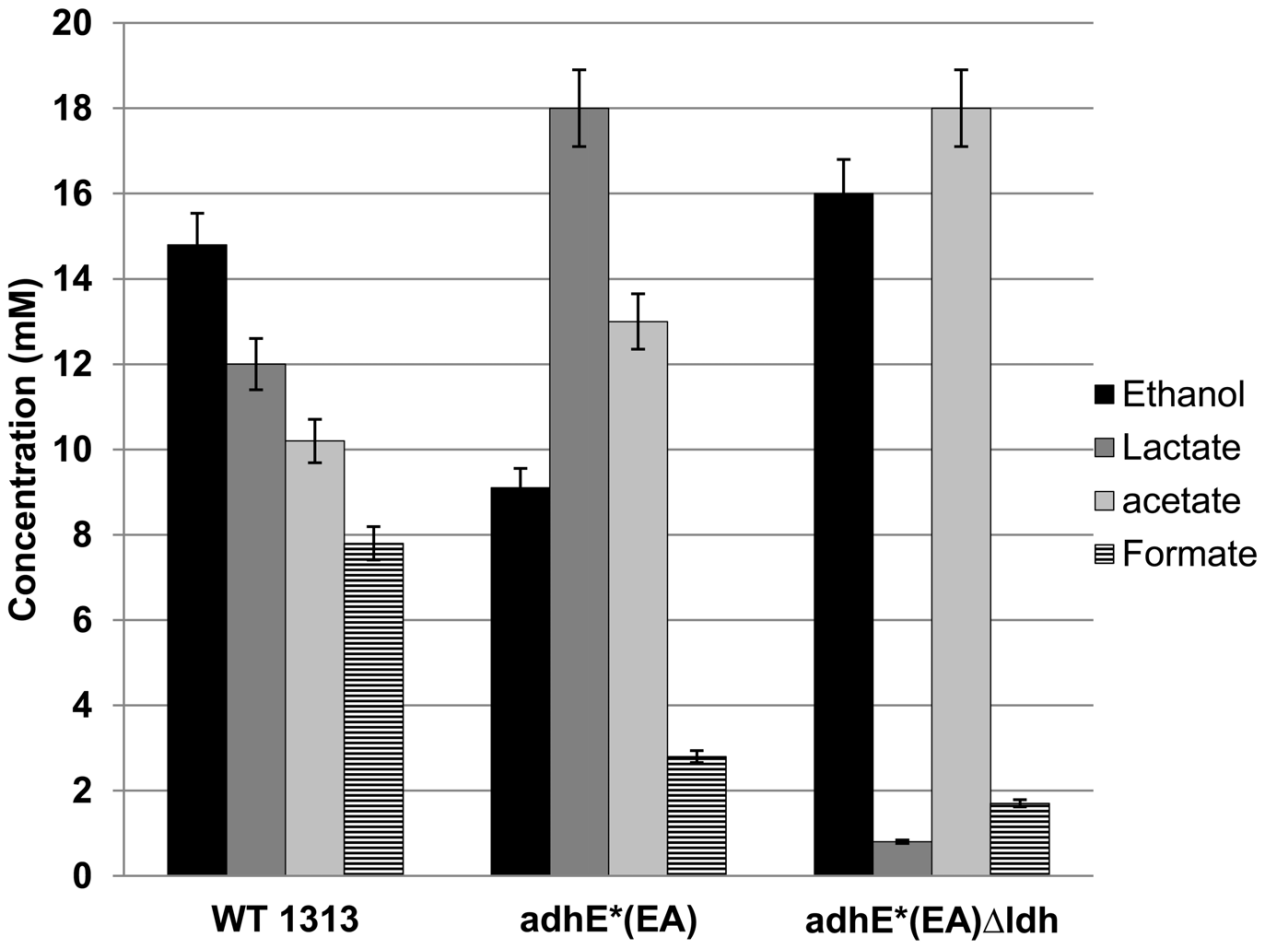
Fermentation products of *C. thermocellum* wild type, C. thermocellum adhE*(EA), and C. thermocellum adhE*(EA) Δ*ldh* on rich medium.

**Figure 3:**
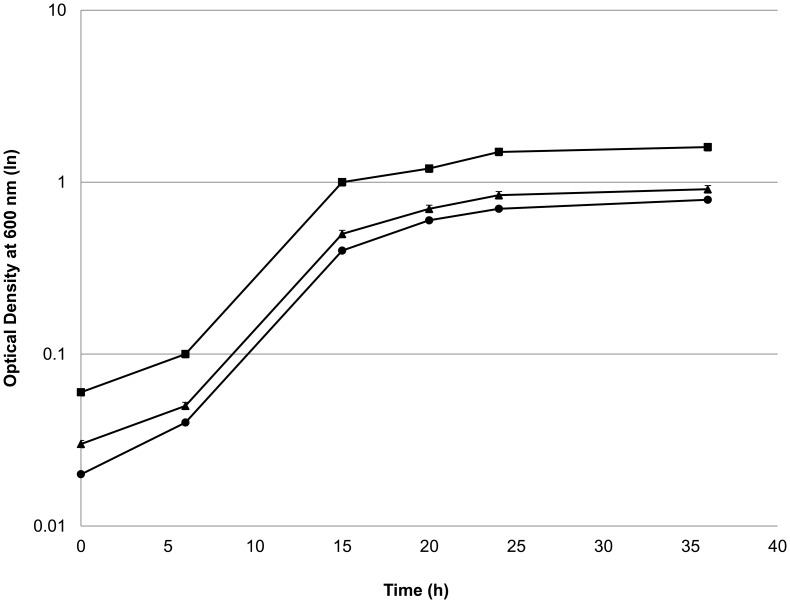
Growth profile of *C. thermocellum* mutants on rich medium. Symbols: closed square, *C. thermocellum* wild type; closed circle, C. thermocellum adhE*(EA); closed triangle, C. thermocellum adhE*(EA) Δ*ldh*.

Consistent with the deletion of *ldh*, production of lactate was nearly eliminated in adhE*(EA) Δ*ldh* strain in both rich and defined media. Ethanol production decreased 40% in the parent adhE*(EA) strain relative to the wild type strain DSM 1313. Deletion of *ldh* restored the carbon flux to ethanol synthesis in rich medium (Fig. 2), increasing ethanol production by ∼78% relative to its parent strain, making it comparable to wild type ethanol production. Acetate production was also higher in adhE*(EA) Δ*ldh*, increasing by 38% and 76% relative to the parent and wild type strains, respectively. While wild type *C. thermocellum* made formate as a fermentation product, adhE*(EA) and adhE*(EA) Δ*ldh* produced 3 and 4.5 fold less, respectively. Interestingly, the growth rate and maximum optical density of adhE*(EA) were decreased as compared to wild type (Fig. 3). Deletion of *ldh* did not substantially rectify this growth defect.

**Figure 4:**
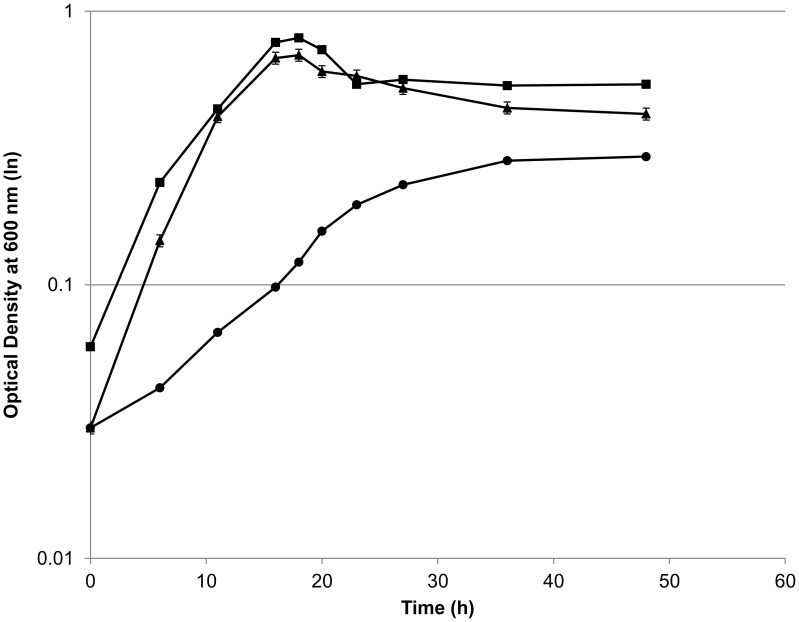
Growth profile of *C. thermocellum* mutants on minimal medium. Symbols: closed square, *C. thermocellum* wild type; closed circle, C. thermocellum adhE*(EA); closed triangle, C. thermocellum adhE*(EA) Δ*ldh*.

**Figure 5:**
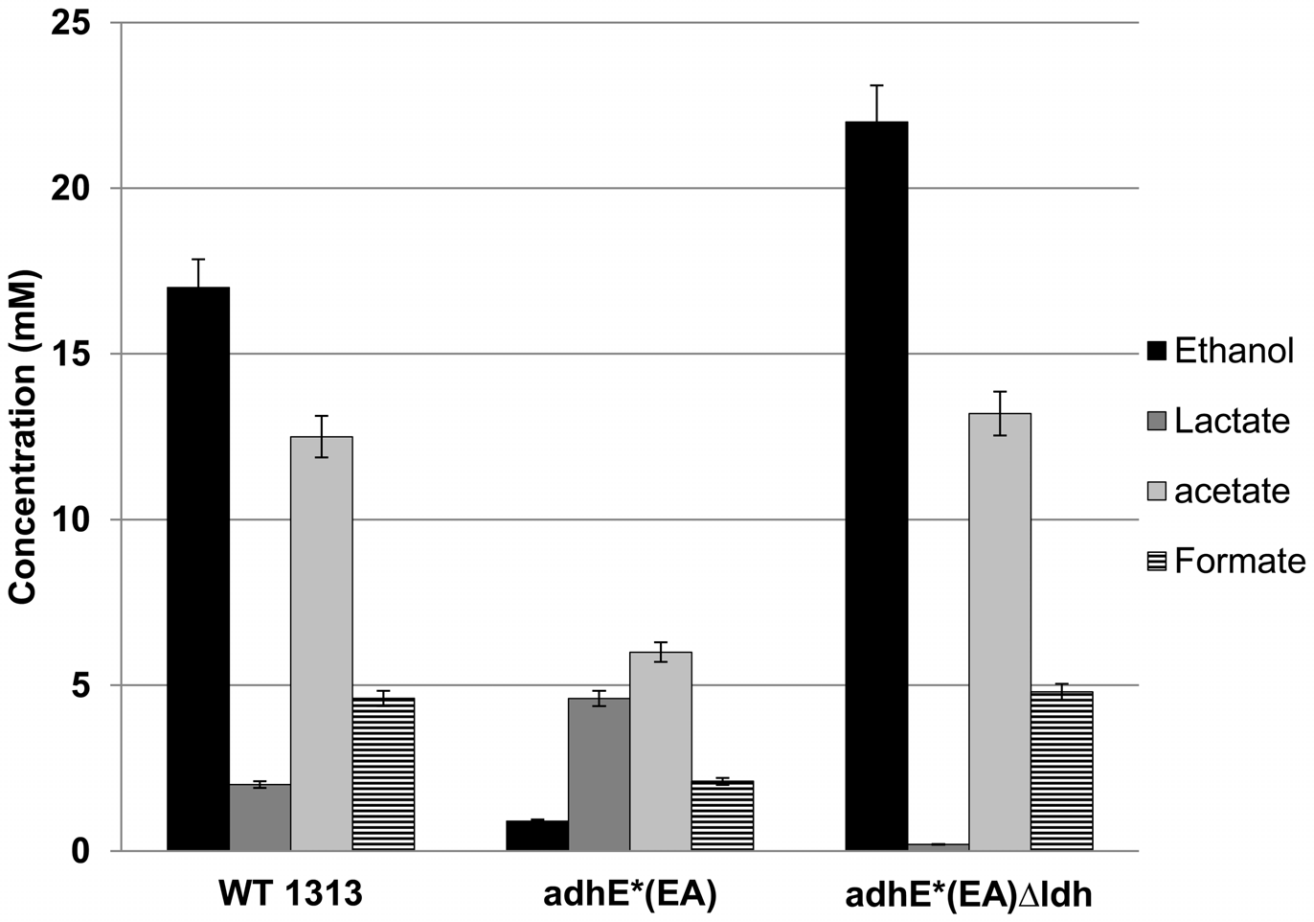
Fermentation products of *C. thermocellum* wild type, C. thermocellum adhE*(EA), and C. thermocellum adhE*(EA) Δ*ldh* on minimal medium.

On minimal medium, strain adhE*(EA) grew very poorly (Fig. 4) and thus made little ethanol. The fermentation profile of adhE*(EA) Δ*ldh*, on the other hand, was much more similar in terms of ethanol, acetate and formate to wild type (Fig. 5). The production of ethanol improved by ∼24-fold compared to the parental adhE*(EA) strain and 30% more than the wild type. Again consistent with the deletion of *ldh*, the presence of lactate was near the limit of detection. In contrast to rich medium, however, adhE*(EA) Δ*ldh* produced similar amounts of formate as wild type during fermentation on minimal medium.

### C. thermocellum adhE*(EA) Δ*ldh* Maintains Ethanol Tolerance Phenotype

**Figure 6:**
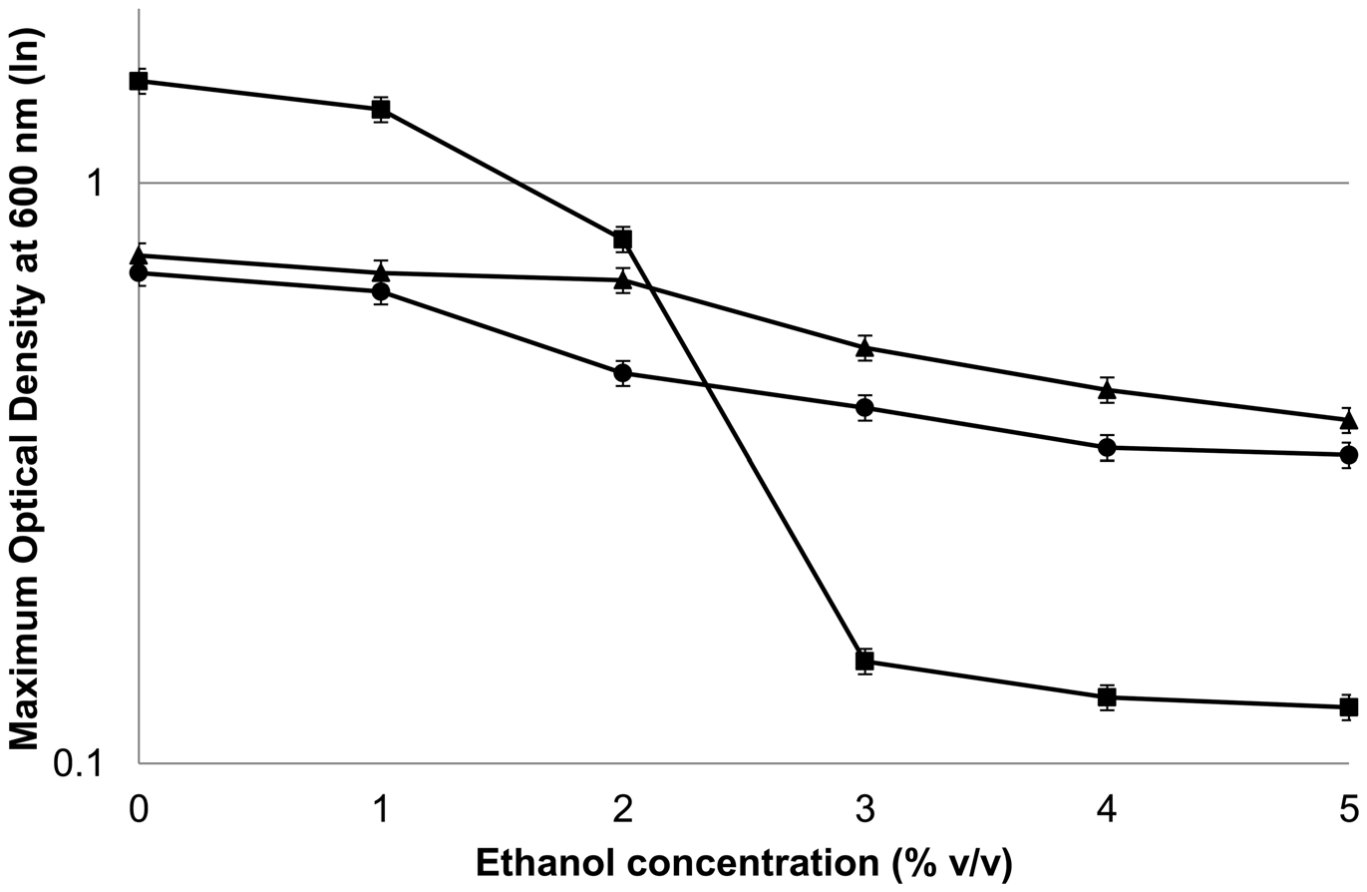
Maximum optical density (OD) attained by wild type and mutant strains of *Clostridium thermocellum* on rich medium supplemented with 0–5% (v/v) added ethanol. Symbols: closed square, *C. thermocellum* wild type; closed circle, C. thermocellum adhE*(EA); closed triangle, C. thermocellum adhE*(EA) Δ*ldh*.

While mutation of *adhE* confers ethanol tolerance, the cause of the deficiency that is corrected by this mutation is unclear. However, the role of AdhE in balancing carbon and electron flux makes it reasonable to suspect that the *adhE* mutation corrects a redox imbalance. Because Ldh is also involved in redox balancing, we wanted to test whether adhE*(EA) Δ*ldh* is still ethanol tolerant. The adhE*(EA) Δ*ldh* strain was evaluated for its growth in rich medium supplemented with ethanol ranging from 0–5% v/v (Fig. 6). The mutant strain adhE*(EA) Δ*ldh* had a growth profile similar to the ethanol tolerant parent strain adhE*(EA) during growth in the presence of exogenous ethanol. While wild type *C. thermocellum* showed a dramatic decrease in its growth yield (maximum OD) above 2% v/v of ethanol, the ethanol tolerant parent strain adhE*(EA) and adhE*(EA) Δ*ldh* continued to show substantial growth even at 5% added ethanol. The maximum OD decreased by approximately half in adhE*(EA) and adhE*(EA) Δ*ldh* as ethanol concentration increased from 0 to 5% v/v.

## Discussion

The mechanism of ethanol tolerance in C. thermocellum adhE*(EA) appears to be related to correcting an imbalance between NADH-NADPH cofactors, perhaps with an overabundance of NADPH being detrimental to growth in the presence of added ethanol [7]. While the predominant pathway for NADPH production in *C. thermocellum* is unclear, possible sources include Rnf (Clo1313_0061–0066) [12], NfnAB (Clo1313_ 1848–1849) [13], or the “malate shunt” consisting of phosphoenolpyruvate carboxylation to oxaloacetate by PEP carboxykinase, NADH-dependent oxaloacetate reduction to malate by malate dehydrogenase, and NADP^+^-dependent malate decarboxylation to pyruvate by malic enzyme [14], [15], [16], [17]. Despite the increased flux to lactate in adhE*(EA), our successful deletion of *ldh* without diminishing ethanol tolerance clearly demonstrates that lactate production is not required as an electron sink in this strain. Instead, removal of Ldh allows flux to ethanol and acetate to continue without the substantial loss of carbon and electrons to lactate production. The low growth rate and yield of adhE*(EA) and adhE*(EA) Δ*ldh* suggests lactate production is a result of overflow metabolism, similar to amino acid production seen previously [18], [19]. Previous work demonstrated that the ethanol:acetate ratio was similar to wild type after deletion of *ldh* in a wild type background [8]. In rich medium, the adhE*(EA) Δ*ldh* strain synthesized 78% more ethanol than parent adhE*(EA) and a similar amount to the wild type. However, acetate production also increased relative to the parent and wild type strains. Interestingly, formate production decreased in both adhE*(EA) strains. In minimal medium, on the other hand, the metabolic effects were different. Ethanol production was ca. 30% higher in adhE*(EA) Δ*ldh* than wild type, but acetate and formate levels were similar between these strains. Additionally, adhE*(EA) Δ*ldh* grew substantially better on minimal medium than parent strain adhE*(EA) for reasons that are not clear. Future investigations into these metabolic differences, including mechanisms to produce NADPH, could help elucidate the mechanisms by which *C. thermocellum* coordinates carbon and electron flux through different pathways.

*C. thermocellum* lactate dehydrogenase (Ldh) is an allosterically regulated enzyme that is activated by the presence of fructose-1,6 bisphosphate (FBP) [20]. In the rich medium, the adhE*(EA) strain synthesized 50% more lactate than the wild type, consistent with a metabolic bottleneck that results in accumulation of FBP. According to this hypothesis, carbon and electron flux would be directed to lactate synthesis and decrease ethanol production. Deletion of *ldh* eliminated lactate as a metabolic outlet for carbon and electrons, but this mutation is unlikely to have corrected the bottleneck that causes FBP accumulation. The similar slow growth rate of adhE*(EA) Δ*ldh* to adhE*(EA) most likely reflects slow flux to acetyl-CoA, and thus to ethanol and acetate.

While the cellulolytic capability of *C. thermocellum* makes it a promising host for production of ethanol via CBP, the low ethanol tolerance of wild type *C. thermocellum* has been a significant hurdle in realizing its potential as an industrial strain. Most studies on ethanol tolerance in *C. thermocellum* have focused on strain ATCC27405, which is challenging to genetically modify. By utilizing a more genetically tractable ethanol tolerant strain of *C. thermocellum*, we have built a platform for further metabolic engineering for high yield and titer ethanol production.

## Supporting Information

File S1 **Plasmid pAMG231 sequence.**
(TXT)

## References

[1] U.S. Department of Energy (2011) U.S. Billion-Ton Update: Biomass Supply for a Bioenergy and Bioproducts Industry. Perlack RD, Stokes BJ, (Leads), Oak Ridge National Laboratory, Oak Ridge, TN. 227.

[2] Lynd LR, van Zyl WH, McBride JE, Laser M (2005) Consolidated bioprocessing of cellulosic biomass: an update. Curr Opin Biotechnol 16: 577–583. 16154338 10.1016/j.copbio.2005.08.009

[3] Lynd LR, Weimer PJ, van Zyl WH, Pretorius IS (2002) Microbial cellulose utilization: fundamentals and biotechnology. Microbiol Mol Biol R 66: 506–577. 10.1128/MMBR.66.3.506-577.2002 PMC120791 12209002

[4] Herrero AA, Gomez RF (1980) Development of ethanol tolerance in *Clostridium thermocellum*: effect of growth temperature. Appl Environ Microbiol 40: 571–577. 7425617 10.1128/aem.40.3.571-577.1980 PMC291623

[5] Shao X, Raman B, Zhu M, Mielenz JR, Brown SD, et al (2011) Mutant selection and phenotypic and genetic characterization of ethanol-tolerant strains of *Clostridium thermocellum* . Appl Microbiol Biotechnol 92: 641–652. 21874277 10.1007/s00253-011-3492-z

[6] Williams TI, Combs JC, Lynn BC, Strobel HJ (2007) Proteomic profile changes in membranes of ethanol-tolerant *Clostridium thermocellum* . Appl Microbiol Biotechnol 74: 422–432. 17124583 10.1007/s00253-006-0689-7

[7] Brown SD, Guss AM, Karpinets TV, Parks JM, Smolin N, et al (2011) Mutant alcohol dehydrogenase leads to improved ethanol tolerance in *Clostridium thermocellum* . Proc Natl Acad Sci U S A 108: 13752–13757. 21825121 10.1073/pnas.1102444108 PMC3158198

[8] Argyros DA, Tripathi SA, Barrett TF, Rogers SR, Feinberg LF, et al (2011) High ethanol titers from cellulose using metabolically engineered thermophilic, anaerobic microbes. Appl Environ Microbiol 77: 8288–8294. 21965408 10.1128/AEM.00646-11 PMC3233045

[9] Tripathi SA, Olson DG, Argyros DA, Miller BB, Barrett TF, et al (2010) Development of *pyrF*-based genetic system for targeted gene deletion in *Clostridium thermocellum* and creation of a *pta* mutant. Appl Environ Microbiol 76: 6591–6599. 20693441 10.1128/AEM.01484-10 PMC2950449

[10] Hogsett D (1995) Cellulose hydrolysis and fermentation by *Clostridium thermocellum* for the production of ethanol. Hanover, NH: Dartmouth College.

[11] Guss AM, Olson DG, Caiazza NC, Lynd LR (2012) Dcm methylation is detrimental to plasmid transformation in *Clostridium thermocellum* . Biotechnol Biofuels 5: 30. 22559230 10.1186/1754-6834-5-30 PMC3536630

[12] Biegel E, Schmidt S, Gonzalez JM, Muller V (2011) Biochemistry, evolution and physiological function of the Rnf complex, a novel ion-motive electron transport complex in prokaryotes. Cell Mol Life Sci 68: 613–634. 21072677 10.1007/s00018-010-0555-8 PMC11115008

[13] Wang S, Huang H, Moll J, Thauer RK (2010) NADP+ reduction with reduced ferredoxin and NADP+ reduction with NADH are coupled via an electron-bifurcating enzyme complex in *Clostridium kluyveri* . J Bacteriol 192: 5115–5123. 20675474 10.1128/JB.00612-10 PMC2944534

[14] Burton E, Martin VJ (2012) Proteomic analysis of *Clostridium thermocellum* ATCC 27405 reveals the upregulation of an alternative transhydrogenase-malate pathway and nitrogen assimilation in cells grown on cellulose. Can J Microbiol 58: 1378–1388. 23210995 10.1139/cjm-2012-0412

[15] Rydzak T, McQueen PD, Krokhin OV, Spicer V, Ezzati P, et al (2012) Proteomic analysis of *Clostridium thermocellum* core metabolism: relative protein expression profiles and growth phase-dependent changes in protein expression. BMC Microbiol 12: 214. 22994686 10.1186/1471-2180-12-214 PMC3492117

[16] Zhou J, Olson DG, Argyros DA, Deng Y, van Gulik WM, et al (2013) Atypical glycolysis in *Clostridium thermocellum* . Appl Environ Microbiol 79: 3000–3008. 23435896 10.1128/AEM.04037-12 PMC3623140

[17] Deng Y, Olson DG, Zhou J, Herring CD, Joe Shaw A, et al (2013) Redirecting carbon flux through exogenous pyruvate kinase to achieve high ethanol yields in *Clostridium thermocellum* . Metab Eng 15: 151–158. 23202749 10.1016/j.ymben.2012.11.006

[18] Ellis LD, Holwerda EK, Hogsett D, Rogers S, Shao X, et al (2012) Closing the carbon balance for fermentation by *Clostridium thermocellum* (ATCC 27405). Bioresource Technol 103: 293–299. 10.1016/j.biortech.2011.09.128 22055095

[19] van der Veen D, Lo J, Brown SD, Johnson CM, Tschaplinski TJ, et al (2013) Characterization of *Clostridium thermocellum* strains with disrupted fermentation end-product pathways. J Ind Microbiol Biotechnol 40: 725–734. 23645383 10.1007/s10295-013-1275-5

[20] Ozkan M, Yilmaz EI, Lynd LR, Ozcengiz G (2004) Cloning and expression of the *Clostridium thermocellum* L-lactate dehydrogenase gene in *Escherichia coli* and enzyme characterization. Can J Microbiol 50: 845–851. 15644899 10.1139/w04-071

